# Behavioral Health Professionals’ Perceptions on Patient-Controlled Granular Information Sharing (Part 1): Focus Group Study

**DOI:** 10.2196/21208

**Published:** 2022-04-20

**Authors:** Julia Ivanova, Tianyu Tang, Nassim Idouraine, Anita Murcko, Mary Jo Whitfield, Christy Dye, Darwyn Chern, Adela Grando

**Affiliations:** 1 School of Human Evolution and Social Change Arizona State University Tempe, AZ United States; 2 College of Medicine University of Arizona Tucson, AZ United States; 3 College of Health Solutions Biomedical Informatics Arizona State University Scottsdale, AZ United States; 4 Jewish Family and Children's Services Phoenix, AZ United States; 5 Partners in Recovery Phoenix, AZ United States

**Keywords:** behavioral health professional, granular information, granular information sharing, electronic health record, integrated health care, electronic consent tool

## Abstract

**Background:**

Patient-controlled granular information sharing (PC-GIS) allows a patient to select specific health information “granules,” such as diagnoses and medications; choose with whom the information is shared; and decide how the information can be used. Previous studies suggest that health professionals have mixed or concerned opinions about the process and impact of PC-GIS for care and research. Further understanding of behavioral health professionals’ views on PC-GIS are needed for successful implementation and use of this technology.

**Objective:**

The aim of this study was to evaluate changes in health professionals’ opinions on PC-GIS before and after a demonstrative case study.

**Methods:**

Four focus groups were conducted at two integrated health care facilities: one serious mental illness facility and one general behavioral health facility. A total of 28 participants were given access to outcomes of a previous study where patients had control over medical record sharing. Participants were surveyed before and after focus groups on their views about PC-GIS. Thematic analysis of focus group output was paired with descriptive statistics and exploratory factor analysis of surveys.

**Results:**

Behavioral health professionals showed a significant opinion shift toward concern after the focus group intervention, specifically on the topics of patient understanding (*P*=.001), authorized electronic health record access (*P*=.03), patient-professional relationship (*P*=.006), patient control acceptance (*P*<.001), and patient rights (*P*=.02). Qualitative methodology supported these results. The themes of professional considerations (2234/4025, 55.5% of codes) and necessity of health information (260/766, 33.9%) identified key aspects of PC-GIS concerns.

**Conclusions:**

Behavioral health professionals agreed that a trusting patient-professional relationship is integral to the optimal implementation of PC-GIS, but were concerned about the potential negative impacts of PC-GIS on patient safety and quality of care.

## Introduction

Though the terms behavioral health and mental health are often used synonymously, the term behavioral health is broader. Mental health focuses solely on a person’s psychological state, whereas behavioral health is a broader umbrella that incorporates physical and mental struggles: eating habits, exercise routines, and alcohol or drug consumption [1,2]. Behavioral care encompasses a variety of health services, including mental health care, psychiatric care, marriage and family counseling, substance use prevention, intervention, treatment and recovery, and others. Behavioral health professionals include, but are not limited to, psychiatrists, psychologists, counselors, clinicians, therapists, social workers, nurse practitioners, and others [3].

Patient-controlled granular information sharing (PC-GIS) allows patients to select “granules” or specific elements of their electronic health records (EHRs) and decide with whom to share this information [4,5]. This paper focuses on the clinical implications of PC-GIS of behavioral health information, long considered to be highly sensitive information by individuals and by US law [2,6-8]. With the advance of integrated physical and behavioral health care delivery, recommendations for expanding patient control of health data have asserted enhanced patient privacy [9-12]. Such suggestions underscore the importance of the PC-GIS process in health care, with patients considering which data to share and which to withhold (eg, “I do not want to share records related to past suicide attempts”). This concept includes the designation of specific data for specific care team members (eg, “I do not want to share past suicide attempt information with my endocrinologist”). Those with a serious mental illness (SMI), defined as an impairment severely interfering with daily activity, are at a higher risk for fragmented care and may, therefore, require different or additional PC-GIS options [13-15]. The literature suggests an evolving tension between patients desiring more access to, and control of, their EHR data and health care professionals who are concerned that such accessibility may negatively impact patient safety, care quality, and cost of care (eg, duplicate labs and diagnostic tests) if critical information is redacted based on patient choice.

Previous studies have established that PC-GIS is attainable using current electronic informed consent systems for care, research, or both, including perspectives on sensitivity and control of information [16-21]. A 394-patient study using granular information sharing for research by Kim et al [17] demonstrated that patients responded positively to granular control, resulting in wide variability of sharing decisions. Caine and Hanania [5] showed that when given the option to exercise granular sharing with various care team participants, all 30 patients chose granular record sharing control over an “all or none” approach. These patients were also most likely (76.7%) to share all information with their primary physicians. A similar 30-patient study found that although PC-GIS on a need-to-know basis (83%) was preferred, patients (20%) admitted they did not understand what items a provider may “need to know” [22]. Soni et al [4,23] evaluated how 25 behavioral health patients would apply PC-GIS using data from their own EHR, comparing patient and health professional sensitivity designation of the same items. Results showed that patients fully (19.3%) and partially disagreed (14.5%) with professionals’ characterization of items. Patient rationale for their sharing choices was complex, including fear of discrimination, perceived relevancy to particular provider disciplines, and trust [4,23]. While these studies emphasize patient perceptions of PC-GIS, they also highlight the need for research focused on health professionals’ perceptions and recommendations on this topic [4,5,16,18,19,22,24,25].

A few studies have explored how health professionals view PC-GIS and how such control may affect clinical care. In a 6-month prospective study by Tierney et al [26], 105 patients in a primary care clinic with 31 professionals were given PC-GIS capability. Of the 24 professionals who completed the poststudy survey, 63% responded “strongly agree” to the statement that patient restriction of information would reduce quality of care, while 54% of those providers agreed that patients having PC-GIS is “OK,” further emphasizing the complexity of PC-GIS [26].

In another study, 20 behavioral health professionals were interviewed about their opinions on PC-GIS and consent [2,15]. Discussion topics were categorized into share, should share, or not share, constituting 100% of professional perceptions. Health professionals noted that patients should share information in cases of medical emergencies (57%), patient history data (52%), and medications and treatments (46%). Health professionals identified certain topics patients seemed reluctant to share: items related to substance use (48%), medical diagnoses (47%), and SMIs (39%) [2]. Overall, the study found that while health professionals agreed patients should have more control over who accesses their EHR (70%), professionals also point out that there is certain information they believe should never be restricted (65%) [15]. Study findings also highlight health professionals’ views that trust and patient comprehension may increase patients’ sharing of information, especially involving sensitive behavioral health information [2,15].

Previous studies suggest that health professionals have mixed or concerned opinions about the process and impact of PC-GIS for care and research [4,23,26,27]. The research in this paper uses the focus group data of 28 integrated health care professionals collected by Ivanova et al in the part 2 of this study [27] to identify changes in opinions and understandings of behavioral health professionals provided with real patient examples and a full case study of PC-GIS. This study hypothesizes that knowing patients’ granular data sharing choices leads to a decrease in behavioral health professionals’ support for PC-GIS. To determine whether such effects occur, this study investigates health professionals’ views on PC-GIS before and after the focus groups and explores potential trends based on cohort differences.

## Methods

### Study Sites and Participants

This study was approved by the Arizona State University Institutional Review Board (No. 00010309). The study sites were two outpatient integrated care facilities using the same EHR platform. One facility treats patients with SMI conditions (SMI facility), while the other predominantly treats patients with general behavioral health (GBH) conditions (GBH facility). This study used definitions from Grando et al [15] for prescribers and nonprescribers. GBH facility professionals were comprised of 15% prescribers and 85% nonprescribers, while SMI facility professionals were comprised of 10% prescribers and 90% nonprescribers.

Four, 2-hour focus groups were conducted at the study sites: two focus groups at each site, with seven behavioral health professionals each. Such a design allows sufficient time for individuals to share their thoughts while providing a small-group environment for conversation [28-30]. Focus group participants were facility employees, 21 years of age or older, who worked closely with patients with behavioral health conditions. Participants from the GBH facility were selected by executive staff, while participants from the SMI facility were recruited using flyers and were self-selected. A representative sample of prescribers and nonprescribers was sought for both facilities.

### Survey

Participants were asked to individually complete a survey prior to the focus group. The survey was adapted from Tierney et al [26] and was comprised of nine statements that were rated on a Likert scale with the following responses: “strongly disagree,” “somewhat disagree,” “neutral,” “somewhat agree,” “strongly agree,” and “don’t know/can’t say” (Table 1) [27]. The survey prompts were divided into six specific aims based on measuring concepts and primary impact, patient or professional (Table 1). After each focus group, participants completed the same survey to evaluate changes in opinions of PC-GIS after seeing how actual patients exercise choices. It was hypothesized that discussion of a demonstrative case study would lead to a decrease in behavioral health professionals’ support for PC-GIS. The survey style (Table 1) lends to measuring opinion changes.

The survey analysis was used to determine the presence or absence of directional opinion change following the focus groups. SPSS Statistics for Macintosh (version 27; IBM Corp) was used for descriptive statistics, Wilcoxon signed-rank testing, Cronbach α tests, scree plots, and exploratory factor analysis on pre- and postsurvey results. Exploratory analysis was done with variables, or groups, of interest with appropriate sample sizes of at least 12 for exploratory factor analysis [31-33]. Descriptive statistics were computed using the bootstrapping option (10,000 replicates), and skewness and kurtosis results were used to confirm normal distribution and, thus, verification of data from the survey [34]. Cronbach α tests were run to ensure multidimensionality of the survey; an α value below .80 was considered evidence of a multidimensional scale [35]. Prompt selections of “don’t know/can’t say” were recorded as blank to avoid skewing results. The Likert scale results ranged from 1.00 (“strong disagreement”) to 5.00 (“strong agreement”). For the pre– and post–Likert scale survey results [35,36], the Wilcoxon signed-rank test (power=0.80, α=.05) was applied to each survey prompt to determine significance for changes after the focus group for all participants (N=28) and between participants serving different patient populations (n=14 each).

Intended response concepts of survey prompts were identified based on results from Tierney et al [26] and were labeled as “prompt aim” (Table 1). Exploratory factor analysis showed which aims, or components, were actively measured by the survey and relationships between prompts based on participant response [36,37]. Varimax rotation was used because survey prompts were not designed to correlate [26]. Measured components of the survey were identified by prompt magnitude in the output component matrix. This analysis revealed the component emphasis from pre- to postsurvey that was used to gauge professionals’ perceptions [35]. Scree plots were used to further validate the component results from the factor analysis where viable components had an exponential slope [35].

**Table 1:** Categorized survey prompts.

Category and prompt aims			Specific survey prompts^a^				
			No.		Phrasing		Directionality
**Patient focused**							
	**Patient control acceptance**						
		2		I am comfortable with patients restricting my seeing some parts of their EHR^b^.		Positive	
		4		I think it is OK for patients to have control over who sees what information in their EHR.		Positive	
		6		It is a good thing for patients to have control over who sees their EHR.		Positive	
	**Patient understanding**						
		1		I believe that patients understand what an EHR is.		Positive	
	**Patient rights**						
		8		The patient owns the information in his or her EHR.		Neutral	
		9		As a patient, I would like to control the information in my EHR that providers can see.		Positive	
**Health professional focused**							
	**Authorized EHR access**						
		3		My patients’ EHRs are viewed only by people who should have access to them.		Positive	
	**Patient-professional relationship**						
		5		Patients preventing me from seeing part or all of their EHR could affect my relationships with them.		Neutral	
	**Quality of care**						
		7		Restricting access to all or part of a patient’s EHR will likely reduce the quality of care I deliver.		Negative	

^a^Survey prompts were grouped by overarching themes and classified as positive, neutral, or negative based on framing. Prompt numbers (eg, prompt 2) refer to chronology of the survey, following placement by Tierney et al [26].

^b^EHR: electronic health record.

### Focus Group

**Figure 1:**
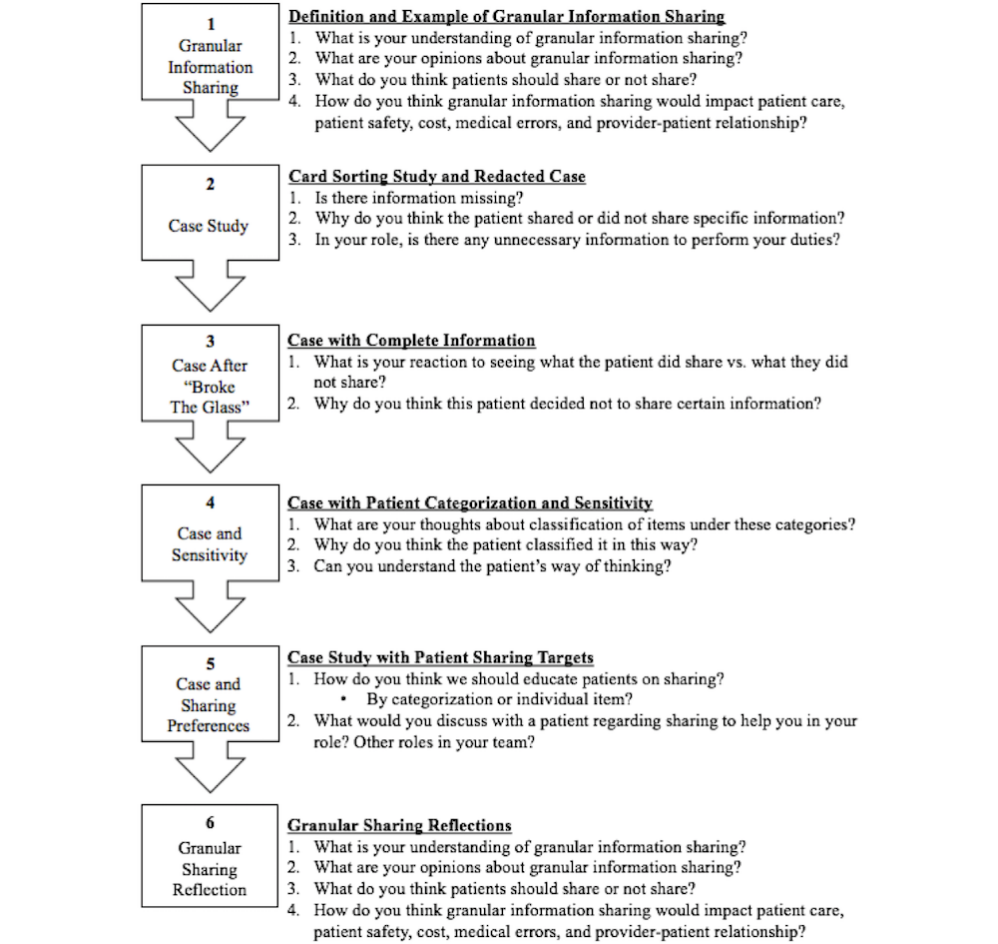
Focus group flow. Section themes are shown on the left with representative questions for each section on the right (numbered). This figure was adapted from Ivanova et al [27].

The focus group was presented in six sections (Figure 1) [27]. In Section 1, baseline perceptions of PC-GIS were elicited using examples and explanations of granular information and sharing [9,17,19,27,38,39]. In Section 2, the Soni et al [4,23], or “card sorting,” study was explained to participants, and a specific case from the study of granular sharing by an actual patient with patient-executed redactions was presented [27]. Participants were then shown the complete patient data set without redaction in Section 3 as if a provider decided to “break the glass,” a term that refers to health care professionals’ retrieval of a patient’s redacted information in an emergency.

During Section 4, the same patient’s data were presented by category (ie, alcohol use and alcoholism, communicable diseases, drug abuse, genetic information, mental health, other addictions, other information, and sexuality and reproductive health) and sensitivity (ie, very sensitive, somewhat sensitive, or not sensitive). Section 5 explored that patient’s choices to share those categories with different health care professionals and institutions.

Finally, in Section 6, participants were asked to reflect on their understanding, opinions, and recommendations for PC-GIS.

Two qualitative analysis techniques were applied to focus group outputs to provide insight into the survey results. Thematic analysis was used to find and define emergent topics of importance to participants [40]. Audio recordings of the focus groups were transcribed through a third party [41] and screened for accuracy by three researchers working sequentially by visual annotation. The validated transcriptions were analyzed using Braun and Clarke’s [42] thematic analysis guidelines and anthropological methodology through six iterations, resulting in quantifiable codes and themes [35]. The units of analysis were meaningful phrases per participant and themes identified through repetition and frequency in transcripts. MAXQDA software (VERBI GmbH) was used to identify and define emerging themes from transcripts. Themes and codes were defined and refined iteratively by three researchers.

### Survey and Focus Group Integration

The exploratory factor analysis results from the survey were used to organize emergent themes and subthemes from the focus group thematic analysis in the last iteration. This step permitted complementary analysis of qualitative and quantitative results.

After providing descriptive statistics and exploratory factor analysis results, themes and codes were used to interpret these results and provide insight into opinion shifts regarding PC-GIS. To compare themes between patient populations, quote matrices and complex coding queries (intersection-set) were performed.

## Results

### Demographics

A total of 28 participants were recruited (Table 2). Out of these participants, 5 (18%) prescribers (ie, physicians and nurse practitioners) and 23 (82%) nonprescribers were identified [15]. This was a demographically representative sample of professionals at each site. All 28 participants took the presurvey and 27 (96%) took the postsurvey.

**Table 2:** Participant roles and population representation.

Role type^a^	Participants, n (%)^b^		
	General behavioral health facility (n=14)	Serious mental illness facility (n=14)	Total (N=28)
Counselors	3 (21)	1 (7)	4 (14)
Nurses	1 (7)	2 (14)	3 (11)
Rehabilitation specialists	1 (7)	2 (14)	3 (11)
Case managers	1 (7)	2 (14)	3 (11)
Clinical coordinators	3 (21)	0 (0)	3 (11)
Administrators	0 (0)	3 (21)	3 (11)
Physicians (prescribers)	2 (14)	1 (7)	3 (11)
Nurse practitioners (prescribers)	2 (14)	0 (0)	2 (7)
Peer mentors	0 (0)	1 (7)	1 (4)
Medical assistants	0 (0)	1 (7)	1 (4)
Discharge planners	0 (0)	1 (7)	1 (4)
Social workers	1 (7)	0 (0)	1 (4)

^a^The table, taken from Ivanova et al [27], groups participants by role types (prescribers are indicated) and patient population.

^b^Percentages may not add up to 100 due to rounding.

### Changes in Behavioral Health Professionals’ Perceptions: Survey

Comparison of the pre– and post–focus group survey responses demonstrated two significant changes: (1) change from strong agreement to strong disagreement (mean <2.5, including SE) with patient-focused survey prompts and (2) change from strong agreement to strong disagreement (mean >3.5, including SE) with professional-focused survey prompts (Table 3). Descriptive analysis results provided skewness and kurtosis statistics exhibiting normal distribution of data, a validation of the survey results and usability of participant responses.

Drilling into the specific prompts, as defined in Table 1, patient understanding (prompt 1, *P*=.001), patient-professional relationship (prompt 5, *P*=.006), and patient control acceptance (prompt 6, *P*=.005) demonstrated significant change, with increased concern about patient control. Authorized EHR access (prompt 3, *P*=.03) and patient rights (prompt 9, *P*=.02) also showed significant change toward concern, validating the study hypothesis. Of note, patient-professional relationship (prompt 5) is considered a negatively phrased expression [26,27], providing insight into the postsurvey shift in the quality-of-care response.

**Table 3:** Results of the descriptive statistics for the pre- and postsurveys.

Prompt no.	Prompt aim	Prompt directionality	Presurvey score^a^, mean (SE)	Postsurvey score^a^, mean (SE)	*P* value^b^
1	Patient understanding	Positive	3.5 (0.2)	2.5^c^ (0.2)	.001
2	Patient control acceptance	Positive	3.5 (0.3)	2.3 (0.3)	<.001
3	Authorized EHR^d^ access	Positive	4.5^c^ (0.2)	3.7^c^ (0.3)	.03
4	Patient control acceptance	Positive	3.9^c^ (0.3)	2.2^c^ (0.2)	<.001
5	Patient-professional relationship	Neutral	3.2 (0.3)	4.0^c^ (0.2)	.006
6	Patient control acceptance	Positive	3.8^c^ (0.2)	2.7^c^ (0.3)	.005
7	Quality of care	Negative	3.6 (0.3)	3.9^c^ (0.3)	.16
8	Patient rights	Neutral	3.6 (0.3)	3.3 (0.3)	.40
9	Patient rights	Positive	3.8^c^ (0.3)	3.1^c^ (0.2)	.02

^a^The survey scores ranged from 1.00 (“strong disagreement”) to 5.00 (“strong agreement”).

^b^*P* values were based on the pre- to postsurvey change using the Wilcoxon signed-rank test.

^c^These statistics are strongly within overall agreement (mean >3.5, including SE) or disagreement (mean <2.5, including SE).

^d^EHR: electronic health record.

Presurvey results loaded into four main components: patient control acceptance, professional considerations, patient rights, and patient understanding. In postsurvey loadings, exploratory factor analysis revealed only three components present; patient understanding was now absent (Table 4). After pairing with the descriptive statistics, results suggest that after the focus group, participants became more concerned with patient rights and patient control acceptance and the impact of these aspects on professional matters*,* such as quality of care and patient-professional relationship.

**Table 4:** Pre- and postsurvey exploratory factor analysis loadings.

Prompt no.	Presurvey component, factor analysis loading^a^				Postsurvey component, factor analysis loading^a^		
	Patient control acceptance	Professional considerations	Patient rights	Patient understanding	Patient control acceptance	Patient rights	Professional considerations
1	0.0	0.0	0.1	0.9^b^	0.4	0.8^b^	0.1
2	0.8^b^	0.1	0.0	–0.1	0.4	0.6^b^	0.3
3	–0.1	–0.8^b^	0.4	–0.2	–0.5	0.7^b^	0.4
4	1.0^b^	0.0	0.1	0.1	0.7^b^	0.4	0.3
5	–0.2	0.7^b^	0.2	–0.4	–0.2	0.1	–0.9^b^
6	0.8^b^	–0.2	0.1	0.3	0.8^b^	0.2	0.3
7	–0.1	0.8^b^	0.4	–0.1	–0.4	–0.3	–0.6^b^
8	0.1	0.1	0.9^b^	0.1	0.2	0.8^b^	–0.1
9	0.9^b^	–0.1	–0.1	–0.2	0.8^b^	0.2	0.2

^a^Negative loadings are due to directionality of prompts and are not significant. Scree plot results ensured overall viability of components.

^b^This value is this prompt’s highest absolute loading for this component.

### Behavioral Health Professionals’ Concerns Around PC-GIS: Focus Group

**Figure 2:**
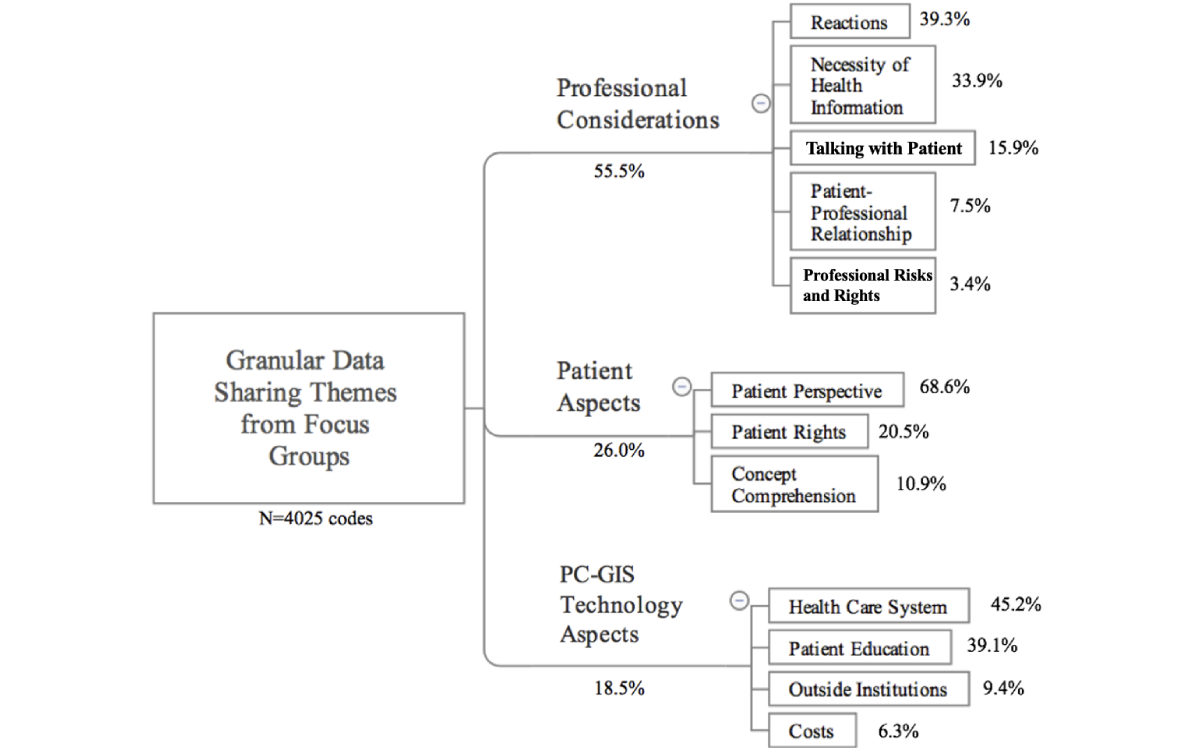
Emergent themes and subthemes from focus group thematic analysis on patient-controlled granular information sharing (PC-GIS). There were a total of 4025 codes.

In the next step of validating the hypothesis, thematic analysis (4025 codes) of focus groups yielded three main themes (Figure 2), complementing the survey components (Table 5). The themes were professional considerations (2234/4025, 55.5%), patient aspects (1046/4025, 26.0%), and PC-GIS technology aspects (745/4025, 18.5%).

The professional considerations theme covers themes that directly impact a provider, including information needed to provide health care services. The patient aspects theme encompasses all topics relating specifically to patient experience and rationale. The PC-GIS technology aspects theme reflects the specific discussion of PC-GIS process and operations (Table 5 and Figure 2).

The survey results show the shift to concern yielding components of patient control acceptance, patient rights, and professional considerations, while thematic analysis shows how professional discussion revolved predominantly around professional considerations, such as necessity of health information (Figure 2). This overall shift toward professional concern around PC-GIS was observed in the focus group discussion and was coded under multiple themes and subthemes; an example quotation is as follows:

...what if there were an issue of depression affecting [the patient’s] hygiene or dental care, and the dentist doesn’t know how to explain that? Similarly, if you had a dentist who was seeing dental care being compromised because you had somebody with an eating disorder, who do they have to collaborate with or even that comfort of making that referral.

Nonprescriber

Similarly, professionals quickly pointed to the complexity of PC-GIS in their domain and the potential for negative impact on patient care:

...I think if a patient has seen numerous doctors, they all should be on the same page with medications because of any contraindications.

Nonprescriber

The survey results reflect understanding and opinions in a quantitative fashion, while the interviews demonstrate how the concepts are linked.

Thematic analysis also conveys the complexity of participant perceptions, with subthemes interweaving patient and professional considerations. Regarding 879 codes, the reactions subtheme included general feedback about PC-GIS (n=255, 29.0%) as well as specific concerns (n=334, 38.0%), predominantly relating to patient safety and health. Patient safety and health encompassed issues ranging from missing medications and the potential for drug-drug interactions to the need for improved physical and mental health integration. A minority (n=149, 17.0%) of participants felt that data sharing in health care as an environment should never be granular: “[This is] not a place to be granular*.*” Other issues surfacing in the health professional concern area included the mismatch of patients’ interpretation of information versus health professionals’ interpretation.

While the patient aspects theme included two subthemes corresponding to exploratory factor analysis components, the major subtheme of patient perspective considered drew greatly on health professionals’ thoughts on patients’ reasoning to share or not share health information. Indeed, 65.0% of 722 codes (n=469) within the patient perspective considered subtheme were specifically related to patient reasoning to not share, such as patient fears or fear of discrimination (n=113, 24.0%). Many instances of patient reasoning (n=201, 42.9%) dealt with patients’ understanding and comprehension.

To provide further context for the quantitative results, participant opinions from within the reactions subtheme (Section 2 codes: 81/116, 69.8%; Section 4 codes: 35/116, 30.2%) were divided into three groups: concerned, supportive, and mixed opinions. For Section 2 questions, regarding the redacted case study, 46% (37/81) expressed mixed opinions, 36% (29/81) were concerned, and 19% (15/81) were supportive. Within Section 4 questions, regarding patient categorization and sensitivity, 43% (15/35) expressed concerned opinions, 37% (13/35) expressed mixed opinions, and 20% (7/35) were supportive. Of note, PC-GIS unease revolved around patient safety:

After you see it in action [Soni et al case study], seeing what they shared versus what was in the chart, I think this safety risk is extremely high.

Nonprescriber

To that end, participants suggested prioritizing a risk-benefit analysis tool for patients as an adjunct to professional-patient PC-GIS interactions:

...if there can be something figured out...just like the duties to warn, just like this that there is some way that you can obtain that information under certain circumstances...I can see where it could actually improve providers’ relations where [the patients are] not going in paranoid that they’re judging because, you know, [they] have a mental illness and [professionals] prejudged [them].

Nonprescriber

A common theme among all focus group participants was that role-specific, essential health information access should be considered when granting PC-GIS authority:

I would be worried that the patient doesn’t share the right information with the right provider.

Prescriber

The problem is that “essential” information depends on the circumstances of the patient:

Okay, I think [PC-GIS is] a good thing because I know if I had depression and there was no good reason for my dentist to know that...now if I have an eating disorder and I’m throwing up all the time, that’s going to ruin your teeth. But I would hopefully choose to share that information with my dentist. But it’s my choice I guess and that’s what’s nice.

Nonprescriber

**Table 5:** Definitions of subthemes for coding and quotations.

Themes and subthemes			Definition		Example quotation
**Professional considerations**					
	Reactions	How professionals react to definitions, case examples, questions, etc		“Yeah, I would be worried that the patient doesn’t share the right information with the right provider.” [Prescriber]	
	Necessity of health information	When a professional references the need for pertinent health information at the point of care		“Patients if they did have a mental illness they’re taking psychiatric medications they’re not going to disclose to the PCP^a^ the meds they’re taking, you can’t check for interactions and then just can’t provide good care.” [Prescriber]	
	Talking with patient	How professionals talk with and seek health information from patients		“Just ask what they’re being treated for those conditions, and so what are they taking.” [Nonprescriber]	
	Patient-professional relationship	When linking the patient-professional relationship to granular data sharing		“Kind of going back to [the] gender dysphoria thing...That is also something that I would want to know because I would want you to be comfortable, and so, I’d want to make sure that I’m referring to you how you want to be as and using the name you want to be known by. And I’d want my office to do the same thing. So, that kind of stuff is also important to know too.” [Prescriber]	
	Professional risks and rights	When a professional considers their own personal risks related to granular data sharing		“Yeah, be it the actual patient or the provider, life being put in jeopardy by not having certain information. I’m thinking more than HIV AIDS...” [Nonprescriber]	
**Patient aspects**					
	Patient perspective	When a professional responds from a patient perspective		“I think more how the patient perceives the information is more sensitive. This is more than perception.”^b^ [Nonprescriber]	
	Patient rights	When a professional references federal or state statutes regarding patient rights		“I really think it is hard because I’ve talked to a lot of people who say that their medical doctors don’t understand the behavioral health side. So if they, if it wasn’t affecting their behavioral health or their medical health, then I think they should have the right to not talk about it if they don’t feel comfortable.” [Nonprescriber]	
	Concept comprehension	When a professional refers to a patient’s uninformed or potentially risky data sharing choices		“But I’ve also seen people’s lives be put in jeopardy because maybe, whether it be a paranoia or just not understanding or something, I don’t want anything shared or, like provider six said, we’ve had it—maybe there is a substance abuse issue.” [Nonprescriber]	
**PC-GIS^c^ technology aspects**					
	Health care system	When a professional refers to sharing information for care coordination with others throughout the health care system		“Not necessarily with that topic that I think if a patient has seen numerous doctors, they all should be on the same page with medications because of any contraindications.” [Nonprescriber]	
	Patient education	When a professional describes or references patient education about granular information sharing		“Tell them the reason why we’re asking, the importance of it, and to help them understand why we need the information.” [Nonprescriber]	
	Outside institutions	When a professional refers to external institutions and organizations with legal control over health data sharing (eg, Department of Homeland Security, courts, law enforcement, and Department of Public Safety)		“I think that’s one of the things that a lot of our patients that they have a legal background or on court-ordered treatment, meaning, they are not necessarily wanting treatment, but the court says that they have to. It is a valid reason for them to be a little nervous and stuff, because ‘what are you going to tell, are you just trying to get more information so I can go back to jail...’” [Nonprescriber]	
	Costs	When a professional highlights the fiscal aspects of granular data sharing (eg, costs to patient, institution, and system)		“I was thinking from a cost perspective. Granular information sharing could increase cost because if you don’t give all the information, I could see a provider redoing things that have already began so they can get the information they need to make a good decision. Whereas, if they have that information and knew what the history was, they would know where to start instead of having to start all the time from the beginning.” [Nonprescriber]	

^a^PCP: primary care physician.

^b^All participants in the focus group agreed with this comment.

^c^PC-GIS: patient-controlled granular information sharing.

### Impact of Patient Populations: Survey and Focus Group Integration

Exploration of potential differences between health professionals using descriptive statistics and drilling down on qualitative data led to identification of two distinct patient populations: GBH and SMI. Descriptive statistics and Wilcoxon signed-rank tests were performed separately for each group (Table 6). Differences were observed in the presurvey, where participants treating an SMI population showed agreement with all positively phrased prompts. Meanwhile, those treating the GBH population showed agreement only with prompts 3 and 7: authorized EHR access (positive phrasing) and quality of care (negative phrasing). Differences were compounded in the postsurvey, where participants treating an SMI population showed agreement only with prompt 5: patient-professional relationship (neutral phrasing perceived as negative) [26,27]. Those treating a GBH population agreed with the negatively perceived prompts (ie, patient-professional relationship and quality of care) and disagreed with a positively worded prompt (ie, patient control acceptance; Table 6). Participants treating an SMI population initially perceived PC-GIS positively, then made a significant shift to neutral or mixed opinion with concern over the patient-professional relationship prompt (*P*=.007). The participants treating a GBH population showed concern over PC-GIS with a shift to concern over the following prompts: patient control acceptance (prompt 2, *P*=.007; prompt 4, *P*=.009), quality of care (*P*=.01), and patient understanding (*P*=.03).

**Table 6:** Descriptive statistics by predominant patient population.

Prompt no.	Prompt aim	Prompt directionality	Presurvey score^a^, mean (SE)			Postsurvey score^a^, mean (SE)			
			GBH^b^	SMI^c^	GBH		*P* value^d^	SMI	*P* value^d^
1	Patient understanding	Positive	3.3 (0.3)	3.7 (0.2)^e^	2.3 (0.3)		.03	2.7 (0.3)	.02
2	Patient control acceptance	Positive	3.1 (0.4)	3.9 (0.3)^e^	1.7 (0.3)^e^		.007	3.0 (0.4)	.02
3	Authorized EHR^f^ access	Positive	4.1 (0.4)^e^	4.8 (0.1)^e^	3.8 (0.4)		.47	3.7 (0.4)	.01
4	Patient control acceptance	Positive	3.4 (0.4)	4.5 (0.1)^e^	1.7 (0.3)^e^		.009	2.8 (0.3)	.004
5	Patient-professional relationship	Neutral	3.9 (0.3)	2.5 (0.4)	4.1 (0.3)^d^		.41	3.8 (0.3)^e^	.007
6	Patient control acceptance	Positive	3.4 (0.4)	4.1 (0.2)^e^	2.4 (0.4)		.28	3.2 (0.4)	.16
7	Quality of care	Negative	4.0 (0.3)^e^	3.2 (0.5)	4.4 (0.3)^e^		.01	3.4 (0.4)	.43
8	Patient rights	Neutral	3.6 (0.4)	3.6 (0.4)	2.9 (0.4)		.10	3.8 (0.4)	.72
9	Patient rights	Positive	3.1 (0.4)	4.5 (0.2)^e^	2.4 (0.3)		.19	3.8 (0.3)	.03

^a^The survey scores ranged from 1.00 (“strong disagreement”) to 5.00 (“strong agreement”).

^b^GBH: general behavioral health.

^c^SMI: serious mental illness.

^d^*P* values were based on the pre- to postsurvey change using the Wilcoxon signed-rank test.

^e^These values are in overall agreement (including SE) or disagreement (including SE).

^f^EHR: electronic health record.

Quote matrices were applied to specify differences between GBH and SMI professionals on survey aims, with some subtopics within themes considered when applicable. GBH professionals showed greater frequency of discussing negative reactions (32/44, 73% of codes), positive reactions (14/22, 64%), professional risks and rights (44/67, 66%), and outside institutions (52/69, 75%). SMI professionals more frequently discussed the following subtopics: do not need to know (21/23, 91%), patient-professional relationship (124/180, 68.9%), trust (22/32, 69%), patient aspects (669/1046, 64.0%), and costs (36/46, 78%). These results reflect large differences in frequencies of thematic analysis coded topics. However, while a topic may be suggested, the discourse may not contain an opinion. Therefore, a second layer of qualitative analysis to identify subthemes was performed.

To identify differences in participant perceptions on themes, subthemes, and topics within subthemes, complex coding queries were used to categorize negative, positive, or mixed perception codes. GBH professionals perceived costs and trust negatively, overall (Table 7). SMI professionals referred to professional risks and rights topics with a negative slant, while the other topics were presented in a mixed or positive fashion. The complex coding queries highlight the complexity of the topic and suggest an impact of patient population on subthemes and topics.

**Table 7:** Complex coding query results of topic perceptions.

Topics, themes, and participant perception		Instances of perceptions for each topic by facility, n (%)		
		General behavioral health facility		Serious mental illness facility
**Do not need to know (within the necessity of health information subtheme)**				
	Negative (n=0)	N/A^a^	N/A	
	Positive (n=1)	1 (100)	0 (0)	
	Mixed (n=4)	2 (50)	2 (50)	
**Patient-professional relationship**				
	Negative (n=4)	4 (100)	0 (0)	
	Positive (n=4)	3 (75)	1 (25)	
	Mixed (n=3)	1 (33)	2 (67)	
**Trust (within the patient-professional relationship subtheme)**				
	Negative (n=1)	1 (100)	0 (0)	
	Positive (n=2)	1 (50)	1 (50)	
	Mixed (n=0)	N/A	N/A	
**Professional risks and rights**				
	Negative (n=15)	11 (73)	4 (27)	
	Positive (n=3)	3 (100)	0 (0)	
	Mixed (n=5)	5 (100)	0 (0)	
**Outside institutions**				
	Negative (n=0)	N/A	N/A	
	Positive (n=10)	10 (100)	0 (0)	
	Mixed (n=3)	3 (100)	0 (0)	
**Costs**				
	Negative (n=3)	2 (67)	1 (33)	
	Positive (n=2)	0 (0)	2 (100)	
	Mixed (n=3)	0 (0)	3 (100)	

^a^N/A: not applicable; there were no instances of this perception regarding this topic.

The mixed methodology analysis focused on the differences between SMI and GBH professionals, where SMI professionals displayed lower levels of concern regarding the process of PC-GIS, more frequently citing the following topics: do not need to know (21/23, 91% of codes), patient-professional relationship (124/180, 68.9%), and trust (22/32, 69%).

## Discussion

### Principal Findings

Results show that behavioral health professionals had fewer positive views on PC-GIS after the focus group, with a significant opinion shift toward concern on the following topics: patient understanding (*P*=.001), authorized EHR access (*P*=.03), patient-professional relationship (*P*=.006), patient control acceptance (*P*=.005), and patient rights (*P*=.02). Qualitative methodology supported these results, as themes and subthemes, such as professional considerations (2234/4025, 55.5% of codes) and necessity of health information (260/766, 33.9%), identified aspects of PC-GIS concerns; indeed, participant opinions after viewing the case study without redactions (Section 4) showed increased levels of concern (7% overall change).

Mixed methodology results after the focus group showed concerns that PC-GIS could negatively impact behavioral health professionals’ ability to deliver optimal care. This perception shift was evident in qualitative results from discussions dominated by patient health and safety topics (combined, 60.3% [70/116] of codes of concerned reaction). Our results show that behavioral health professionals remained highly concerned about patient granular control for a variety of reasons [18]. Health professionals in our study highlighted potential negative effects of granular sharing, including impact on the professional-patient relationship and lack of access to necessary health information, reflected in the professional considerations theme. A minority of professionals (149/879, 17.0%) considered health care as simply “not a place to be granular,” while most acknowledged acceptance of the trend toward increasing PC-GIS and offered concrete recommendations for proactive processes that could help ensure patient safety while preserving record sharing choice.

Much of the literature focuses on patient perspectives of PC-GIS, demonstrating that patients respond positively to having granular control over data sharing [5,17,18,24], while casting doubt on a patient’s understanding of information relevancy or professionals’ “need to know” [5,15]. Our study reflects similar concerns by behavioral health professionals, where subthemes included necessity of health information (260/766, 33.9% of codes) and patient concept comprehension (13/115, 11.3%). Our thematic analysis demonstrated that when professionals are shown results of a patient’s granular sharing choices, they view the choices from the patient perspective, while expressing apprehension that necessary role-specific information may not be appropriately shared [4]. Employing shared decision-making using specialty-tailored methods may help alleviate such concerns [5,15,27].

A trusting bond is important in health care delivery and is a continued underlying basis in quality of care and patient outcomes in health literature [2,43-46]. Our qualitative results refer to trust when subthemes such as talking with patient, patient-professional relationship, and patient education directly deal with strengthening the relationship and understanding between health professionals and patients. A recent study by Esmaeilzadeh [47] showed that patients’ trust in providers influences their trust in information sharing technology. Our qualitative results exemplify some processes that health professionals may use to strengthen trust: “Tell them [patients] the reason why we’re asking, the importance of it, and to help them understand why we need the information.” Indeed, this type of approach to strengthening the professional-patient relationship was a common recommendation in our study, as well as in existing literature, to alleviate professionals’ worry over patients not sharing appropriate information [15,27,44,46,47].

Proceeding further into the topic of education, professionals involved in PC-GIS must have the knowledge, background, and tools to assist patients in making safe sharing choices. This is exemplified in the case where a professional supports a patient’s choice to withhold behavioral health diagnoses and medications from a patient’s dentist. In reality, oral health and behavioral health have many important intersections, including substance use disorder and eating disorders [48]. Therefore, organizations and institutions must ensure that their PG-GIS process and professionals are prepared to provide sound advice to ensure patient safety. While health care institutions need to consider PC-GIS use in integrated and coordinated care, attention should be paid to critical policies, such as Title 42 CFR (Code of Federal Regulations) Part 2 and the CARES (Coronavirus Aid, Relief, and Economic Security) Act [8,38,49], and safe implementation of health care technologies relevant to health information exchange and patient EHRs [11,22]. Professionals must be actively engaged in the creation, implementation, and monitoring of data sharing policies that integrate relevant statutes as well as advances in technology and biomedicine. Our results show that when provided with an in-depth explanation of tools affecting health care delivery, health care professionals can begin the necessary dialogue regarding concerns and recommendations for improvement [27]. Identification of concerns for all stakeholders is necessary for successful implementation of health care technologies, such as PC-GIS.

While some research exists that considers health professionals’ opinions on granular sharing and consent, few studies have explored the behavioral health realm, where care generates and uses highly sensitive patient information [26,50,51]. Our study has added to knowledge on behavioral health professionals’ perceptions [27], while exposing divergence between behavioral health professionals who treat differing patient populations. Results comparing behavioral health professionals’ predominant patient populations indicate that those working with an SMI population displayed lower levels of concern and focused on patient aspects (669/1046, 64.0% of codes), as compared to participants working with the GBH population (patient control acceptance, *P*=.004; patient understanding, *P*=.02).

Our results provide a perspective on the relevance of studies from physical health settings application to behavioral and integrated health groups [13]. Based on a pilot study where some patients were given PC-GIS capabilities, Tierney et al [26] showed that 63% of the providers strongly agreed that granular information restriction will likely reduce the quality of care delivered. Our study found that after the focus group intervention, behavioral health professionals agreed overall with Tierney et al’s findings, moving from strong agreement to strong disagreement regarding patient-focused survey prompts. Such outcomes show that behavioral health professionals have similar levels of concern about PC-GIS as compared to other health professionals. However, their concerns may differ in scope and application, as behavioral health patients may be more vulnerable to addictions, discrimination, and influence from outside institutional pressures [52-54].

The divergence observed became more visible when looking at the two facilities. Survey analysis showed that participants from the SMI facility not only viewed PC-GIS more positively than those from the GBH facility, but they also displayed differences in what survey aims (ie, patient-professional relationship and patient rights) we found to have significant change from pre- to postsurvey. Professionals from the SMI facility displayed a significant (*P*=.007) shift from neutral to mixed opinions regarding the patient-professional relationship, an aim falling within the patient aspects category. Results from the quote matrices reinforced these outcomes, as the conversation by SMI facility professionals focused more heavily on patient aspects (669/1046, 64.0% of codes). On the contrary, GBH facility participants showed consistent agreement (Table 6) on professional-focused prompts and disagreement with patient-focused prompts. Health professionals working with an SMI population may view PC-GIS with less concern because they typically interact with their patients more frequently over longer periods of time [55,56]. One approach to alleviating PC-GIS concerns may be in bolstering patient-professional relationships and communication.

Integrated care that emphasizes transparency and patient-centeredness are health system goals [9,10]. This study highlights key aspects of PC-GIS that must be considered for its broader and deeper integration in the care environment. Improving process transparency benefits patients and professionals by exposing gaps in care, differences in patient safety and outcomes, and drivers of costs [2,9,15,18,57]. Understanding diverse professional perspectives is critical in developing granular consent systems that balance patient-professional information needs. Results of this study will be used in the development of a PC-GIS tool, My Data Choices, to allow patients with behavioral health conditions to choose which medical records (eg, mental health information) to share with whom (eg, behavioral health providers) and for what purpose (eg, health care).

### Limitations and Future Research

While our study had a small number of participants, it was a demographically representative sample within each integrated care study site, capturing the essence of the integrated health care team and their role-inspired concerns and needs. The sample size may affect the study replicability; further research with a larger sample size and in a variety of behavioral health and integrated care settings is needed. Another limitation in our study is the pragmatic difference in recruitment methods to accommodate facility leadership preference. Future work may consider semistructured interviews to more clearly identify differences in health professionals’ needs. Finally, our study presented a single exemplar case derived from Soni et al [4,23]. Future studies could leverage additional patient data sharing scenarios.

### Conclusions

This study enhanced what is known about PC-GIS by systematically exploring the rationale behind behavioral health professionals’ perceptions, using results from a study of PC-GIS by real patients using their own data. Outcomes show that as health care professionals learn about PC-GIS implementation, they develop greater levels of concern. However, professionals balanced their concern with material recommendations for PC-GIS process improvement that ranged from patient and staff education to strengthening patient trust. Participants agreed that an informed and transparent system for health information sharing is needed to foster the mutual trust required to implement robust PC-GIS.

## Abbreviations

CARES: Coronavirus Aid, Relief, and Economic Security
CFR: Code of Federal Regulations
EHR: electronic health record
GBH: general behavioral health
PC-GIS: patient-controlled granular information sharing
SMI: serious mental illness
